# Effect of low frequency repetitive magnetic stimulation at Shenmen (HT7) on sleep quality in patients with chronic insomnia

**DOI:** 10.1097/MD.0000000000021292

**Published:** 2020-07-24

**Authors:** Jie Yuan, Hui Wang, Jie Chen, Yaling Lei, Zhaoxin Wan, Yuan Zhao, Zucheng Han, Dongling Liu, Pei Wang, Fan Luo, Yuan Wang, Yue Cao

**Affiliations:** a School of Basic Medical Sciences, Chengdu University of Traditional Chinese Medicine, Chengdu; b Department of Encephalopathy, Shaanxi Provincial Hospital of Traditional Chinese Medicine; c Syndrome and Treatment Research Office For Insomnia With Yin Yang Sequential Treatment, Shaanxi Administration of Traditional Chinese Medicine; d Department of Geriatrics, Xi’an Hospital of Traditional Chinese Medicine, Xi’an, China.

**Keywords:** chronic insomnia, HT7, insomnia severity index, randomized controlled trial, right dorsolateral prefrontal cortex, repetitive magnetic stimulation, resting motor threshold, repetitive transcranial magnetic stimulation, **s**tudy protocol

## Abstract

**Background:**

Insomnia is a common, recurrent, and tenacious sleep problem, especially the chronic insomnia. Repetitive transcranial magnetic stimulation (rTMS) at right dorsolateral prefrontal cortex (r-DLPFC) is used in chronic insomnia, and repetitive magnetic stimulation (rMS) at Shenmen (HT7) acupoint may be an alternative approach. However, the efficacy and mechanism of rMS at HT7 acupoint for chronic insomnia has not been confirmed.

**Methods/design:**

This is a 3-armed randomized positive-controlled noninferiority clinical trial. We will allocate 45 subjects aged between 18 and 65 years old, diagnosed with initial chronic insomnia over 3 months to 3 groups randomly in a ratio of 1:1:1. Patients in the experimental group will be treated with rMS at HT7 acupoint while the others in the control group will be treated with rTMS at r-DLPFC or waiting treatment. All will be given rMS at HT7 or rTMS at r-DLPFC or no treatment for 10 days, and then received 20-day follow-up. Patients will be evaluated with the insomnia severity index and Pittsburgh sleep quality index for sleep state, Beck Depression Inventory-2nd edition scores for the depression state, Beck anxiety inventory scores for the anxiety state, and Montreal Cognitive Assessment scores for the cognition state before and the 10th day of treatment, 30th day of follow-up; study on mechanisms of rMS will be revealed through the resting motor threshold diversity of the motor cortex before and the 10th day of treatment, 30th day of follow-up. Baseline characteristics of patients will be summarized by groups and compared with Chi-squared for categorical variables, and analysis of variance or Kruskal–Wallis test for the continuous variables. Primary and secondary outcomes according to the measurement times are applicable to univariate repetitive measurement deviation analysis or analysis of variance, or Kruskal–Wallis test.

**Conclusion:**

The present study is designed to preliminarily investigate short-term efficacy and mechanism of rMS at HT7 acupoint therapy on chronic insomnia, also to explore the correlation between motor cortex excitability and chronic insomnia. With this research, we are looking forward to find out an appropriate alternative and easy therapy for chronic insomnia individuals compared with rTMS at r-DLPFC.

**Trial Registration:**

The trial was registered on Chinese Clinical Trial Registry (http://www.chictr.org.cn/index.aspx) with the ID ChiCTR1900026844 on October 24, 2019.

## 1 Introduction

Insomnia is a common, recurrent and tenacious sleep problem, adults incidence in the range of 10% to 15%, especially the chronic insomnia (CI).^[1]^ CI has been regarded as an independent risk factor for coronary heart disease, acute myocardial infarction, heart failure, hypertension, diabetes, depression, dementia, and other chronic diseases. Besides, it may cause daytime fatigue, cognitive impairments (attention, memory, executive ability), negative emotions, and affect the individuals quality of life.^[2–7]^ It indicates that hyperarousal is one of the main pathologic mechanisms of CI.^[8]^

The short-term efficacy of sleep agents has been proved, but they often lead to a series of side effects, like daytime dysfunction, sleep driving, abnormal behavior, and depression deterioration. Moreover, it does exist addiction, which might make patients resistance and low compliance for long-term users.^[9,10]^ Due to the lack of cultural background and diplomats in China, the implementation of cognitive behavioral therapy in basic hospitals and general hospitals is rarely used at present. Thus, it is essential to select an effective, safe, no side effects, and no addiction sleep method. Repetitive transcranial magnetic stimulation (rTMS) is a noninvasive neuroregulatory technique in line with the above characteristics, which has been widely used in clinic by reducing cortical excitability in CI patients.^[11]^ Low-frequency (1 Hz) rTMS at bilateral frontal or parieooccipital regions is recommended for sleep disorders in the *Chinese experts consensus on rTMS* (level of evidence: level II/III).^[12]^ However, the rTMS for patients with intracranial metal implants, stents, glaucoma, and hypertension is limited. In traditional Chinese medicine (TCM), insomnia is an appropriate disease in the acupuncture spectrum of diseases. *Shenmen* acupoint (HT7) is the *yuan* acupoint of Heart-Channel, the most frequently selected for insomnia.^[13]^ Acupuncture at HT7 acupoint can activate related brain regions highly associated with improving sleep, emotional, and cognition.^[14,15]^ Thus, the problem of acupuncture therapy is that its manipulations are difficult to replicate, and some patients cannot tolerate repeated pain stimulation. Studies have shown that the effects of repetitive magnetic stimulation (rMS) at acupoints can be quantified, similar to acupuncture effect,^[16]^ noninvasive and painless. In particular, low frequency (1 Hz) rMS at HT7 acupoint can make brain activity in good order and reduce brain excitability for the healthy.^[17]^ Furthermore, low frequency (1 Hz) rMS at HT7, PC6 and SP6 can be also good for the subhealth insomnia ones in enhancing interaction between brain regions, increasing transmission speed and efficiency between brain networks, and improving brain cognitive function.^[18]^ However, there are few clinical studies on low frequency (1 Hz) rMS at HT7 acupoint for CI.

However, compared with rTMS at right dorsolateral prefrontal cortex (r-DLPFC), no sufficient clinical trial data and solid evidence could confirm whether acupoint could yield certain efficiency in treating CI and how it works. It is necessary to verify the effectiveness and mechanism of rMS at acupoint on the sleep quality and cortical excitability from senior quality studies. Now, the evidence about low-frequency rMS at HT7 acupoint for CI is inadequate and must apply further strictly design clinical studies.

The primary objectives of this study are to evaluate the short-term efficacy and mechanism of rMS at HT7 acupoint and follow-up on CI preliminarily.

## 2 Methods/design

### 2.1 Study design

This is a single-center, randomized, positive-controlled, noninferiority clinical pilot trial. A total of 54 participants will be randomized into 3 groups at a ratio of 1:1:1. Participants in the treatment group will be treated with rMS at HT7 acupoint. Participants in the control group will be treated with rTMS at r-DLPFC or waiting treatment.

**Figure 1:**
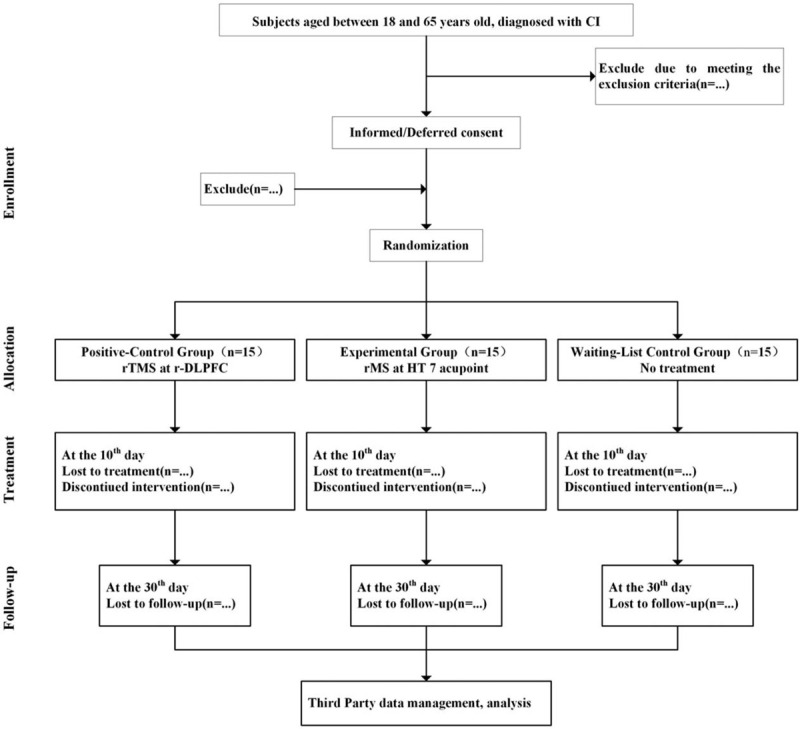
Flow of subjects randomized to receive repetitive magnetic stimulation (rMS) at HT7 or repetitive transcranial magnetic stimulation (rTMS) at right dorsolateral prefrontal cortex (r-DLPFC) or waiting treatment.

The whole study period is 35 days, including 5-day baseline observation, 10-day treatment, and 20-days follow-up. Subjects will be evaluated with scales for clinical efficacy before and the 10th day of treatment, 30th day of follow-up, and monitored by rMS/rTMS for therapeutic mechanism before and the 10th day of treatment, 30th day of follow-up (Fig. 1).

The clinical trial results will be reported according to the Standards for Reporting Interventions in Clinical Trials of Acupuncture statement.^[19]^

### 2.2 Ethics

This study has been approved by the ethics committee of Shaanxi Province Hospital of traditional Chinese Medicine ([2019] ethical review No. 08), and registered on http://www.chictr.org.cn/index.aspx with the ID ChiCTR1900026844. Informed consents will be obtained from all the subjects involved. Any modification of the study protocol or informed consent that may affect the rights and interests of the participants or the implementation of the study shall be reported to the Ethics Committee for approval. In case of any serious adverse event in the trial, the ethics committee shall promptly review it and recommend written modifications, including sufficient authority to suspend the trial.

### 2.3 Study population

#### 2.3.1 Inclusion criteria

1. Subjects, diagnosed with primary insomnia, insomnia severity index (ISI) scores >7 points,^[20]^ according to the Diagnostic and Statistical Manual of Mental Disorders-Fifth Edition (DSM-V), F51.01^[21]^;
2. Subjects, diagnosed with initial CI (course of the disease ≥3 months and frequency ≥3 times/week)^[8]^;
3. Subjects, aged between 18 and 65 years old, right-handed, male or female;
4. Cooperation during examination, without aphasia and severe cognitive disorders;
5. Sign the informed consent.

#### 2.3.2 Exclusion criteria

1. Subjects, diagnosed with secondary insomnia, such as mental disorders (depression, anxiety, schizophrenia, diphasic, etc) or physical diseases (pain, stroke, coronary heart disease, tumor, etc) or other nonpathologic factors (poor sleep hygiene, jet lag, shift work, etc);
2. With severe depression and anxiety (PHQ-9 scores ≥20 points; GAD-7 scores ≥19 points),^[22]^ high risk of sleep apnea (>3 items in the Stop Bang questionnaire), aphasia and severe cognitive dysfunction (MMSE scores <17 points)^[23]^;
3. With severe heart disease, malignancy, kidney, and liver function insufficiency;
4. Dependence or abuse of alcohol or drugs;
5. With metal foreign bodies (cochlear implant, built-in pulse generator, aneurysm clip, stent, etc) at the treatment sites within 30 cm depth under the skin;
6. Participation in any other clinical trial or taking benzodiazepines in recent 2 weeks or receiving TMS, transcranial direct current stimulation, electroconvulsivetherapy, acupuncture and other treatments regularly over the last 1 month;
7. Pregnant or lactating women.

#### 2.3.3 Withdrawal criteria

1. Violation of the inclusion criteria and clinical trial protocol or fulfillment of the exclusion criteria;
2. Do not receive 10 consecutive treatment days or receipt of <20 rMS sessions;
3. Serious adverse events;
4. Loss to follow-up;
5. Withdrawal of consent by the subject or a legal representative;
6. Self-replacement of the treatment package that could affect the study results without permission from the investigator.

### 2.4 Study settings and recruitments

The present study will be conducted in the Shaanxi Province Hospital of Traditional Chinese Medicine.

Participants with the diagnosis of CI will be recruited at above-mentioned hospital, who mainly come from hospital wards and clinics. Besides, posters, leaflets, and the routine free clinics will be also helpful. Sure, except these, the hospital websites, WeChat, and Microblogging are powerful advocacy media.

### 2.5 Study group

Forty-five participants who meet the criteria with informed consents will be selected. The subjects will be randomly divided into 3 groups in a 1:1:1 ratio, namely the experimental group, positive-control group and waiting list control group, 15 cases in each group.

### 2.6 Study time

This clinical study will be conducted from August 1, 2019 to August 1, 2021.

### 2.7 Interventions

Interventions are selected base on the theory of TCM and expertise. The physicians involved have received their practitioners’ license from China's National Health and Family Planning Commission as well as acupuncture and rTMS clinical skill training, who have worked for more than 3 years. Subjects in experimental group will receive rMS at HT7, and others in control group will receive rTMS at r-DLPFC or waiting treatment. Besides, if the symptoms were severe or do not improve during the treatment or the waiting period, Eszopiclone tablets (3 mg Qn) would be allowed.

#### 2.7.1 Experimental group

##### 2.7.1.1 rMS at HT7 acupoint

**Figure 2:**
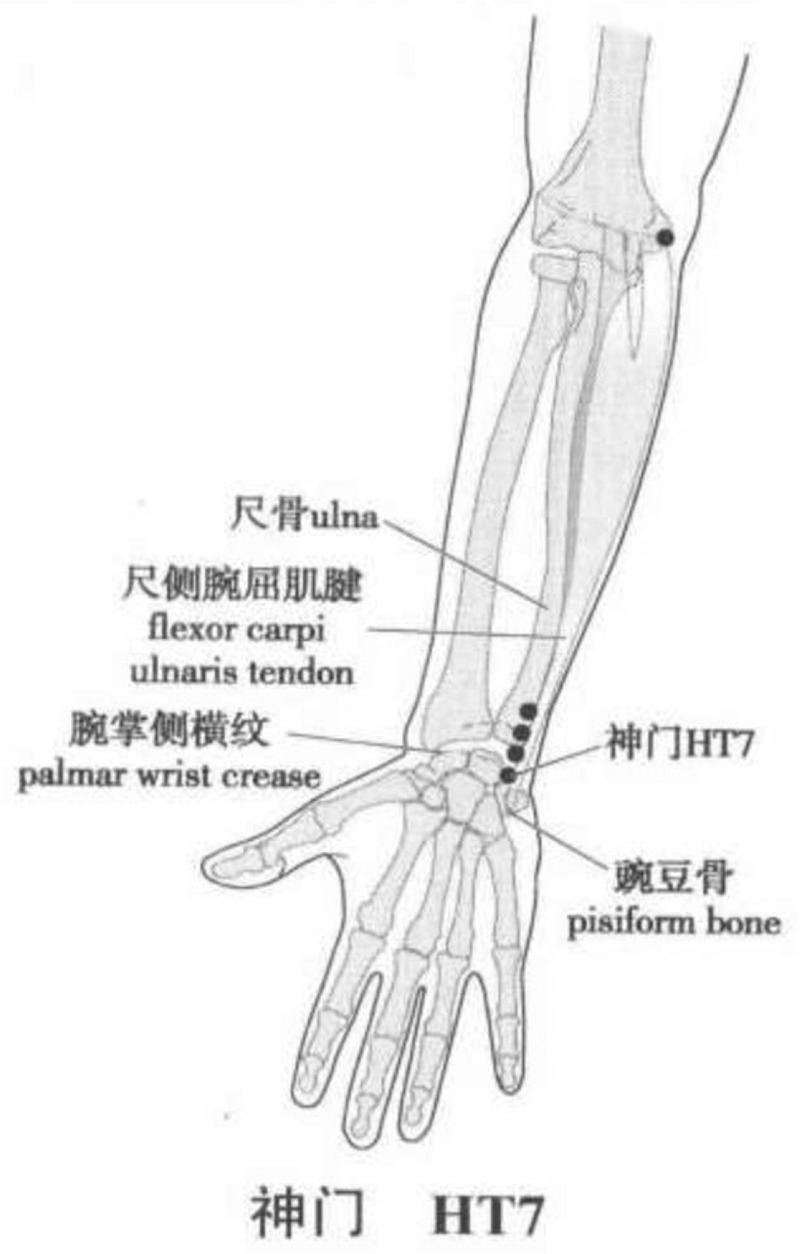
Location of Shenmen (HT7) Acupoint. Longxiang H. WHO Standard Acupuncture Point Locations in the Western Pacific Region (Chinese-English bilingual edition). Beijing: People's Medical Publishing House, 2010.

The rTMS therapeutic apparatus (CCY-II model; Wuhan YiRuiDe Medical Supplies New Technology Co Ltd, Wuhan, China) were used. rTMS is applied to treat CI according to the modern neuro-rehabilitation theory. Acupoint is only left HT7 (*shenmen*) acupoint according to the TCM theory. After preparation, test the resting motor threshold (RMT): the participants will take the sitting position, keep the mood stable and the muscles relaxed. During stimulating M1 region, the coil will be tangent to the scalp, and it will be advisable to adjust the coil to get the maximum amplitude and the best repeatability of motor evoked potential. The stimulus intensity will begin from 30% and gradually increase at a rate of 2.5%. The minimum stimulus intensity (RMT) will be the abdominal contraction in the abductor pollicis brevis muscles of contralateral thumb in 5 out of 10-times consecutive stimuli; locate the stimulus area: the stimulation coil will be placed at the left HT7 acupoint (on the anteromedical aspect of the wrist, radial to the flexor carpi ulnaris tendon, on the palmar wrist crease) (Fig. 2); set the stimulus parameters: the standard mode is selected as the stimulus mode, for instance the frequency as 1 Hz, the total pulses number as 1800, and the intensity as 25% RMT.^[24]^ Start the stimulation after checking the above parameters, and then remove the coil flap after the stimulation. Participants will be treated 2 sessions per day for 10 consecutive days.

#### 2.7.2 Positive-control group

##### 2.7.2.1 rTMS at r-DLPFC

Low-frequency (1 Hz) rTMS at r-DLPFC is recommended for sleep disorders in the *Chinese experts consensus on rTMS* (level of evidence: level II/III)^[12]^ and *Guidelines for The Diagnosis and Treatment of Insomnia in China*.^[25]^ After preparation, test RMT: the measurement is same as the experimental group; locate the stimulus area: the stimulation coil will be placed tangent to the scalp on the r-DLPFC (8 cm in front of the midpoint of the connection between 2 ear tips, and then 6 cm toward the right side); set the stimulus parameters: the intensity is selected 100% RMT. Other parameters are same as the experimental group. Participants will be treated 2 sessions per day for 10 consecutive days.

#### 2.7.3 Waiting-list control group

The participants in the waiting group will not be treated within 10 days during the study period, and then treated same as the positive control group from the 11th day.

### 2.8 Adverse events observation

It could be that patients with epilepsy or headache, scalp discomfort, and tinnitus at stimulating are more likely related to rTMS. Regional skin numbness around the HT7 acupoint occur in patients is probably connected to rMS at HT7 acupoint. With the patients’ diary, all adverse events are to be recorded and measured during treatment and the follow-up period. Serious adverse events should be reported to the principal investigator immediately. All details will be documented, coil location and stimulus intensity adjustment (avoid the more sensitive orbitofrontal cortex, 100% RMT → 80% RMT), as well as appropriate medical care will be given at once. For the participants with severe CI during study, they would be administered with Eszopiclone tablets (3 mg Qn). However, if the subjects cannot tolerate the 80% RMT intensity and/or take Eszopiclone tablets more than twice a week, they will be considered as drop outs.

### 2.9 Primary outcomes

The ISI scores for the severity of CI before and the 10th day of treatment, 30th day of follow-up. The RMT for excitability of the motor cortex before and the 10th day of treatment, 30th day of follow-up.

### 2.10 Secondary outcomes

Pittsburgh sleep quality index^[26]^ scores for the sleep quality of CI patients, Beck Depression Inventory-2nd edition scores for the depression state, Beck anxiety inventory scores for the anxiety state, and Montreal Cognitive Assessment scores for the cognition state before and the 10th day of treatment. Patients’ diary for tracking their mood, sleep, diet, adverse events, and others during the research. All the dropouts and causes will be documented in case report form.

### 2.11 Estimation of sample size

This study is just a pilot study, which designed for efficacy evaluation and mechanism investigation of targeted therapy preliminarily before and after treatment as well as follow-up period. We will plan to enroll a total of 45 participants in this study.

### 2.12 Randomization

After recruitment, the patients who meet the eligibility criteria would be randomly and equally divided into 3 groups at a ratio of 1:1:1 with random number table method. Each subject will be registered with a unique ID. After the clinical screening and getting the informed consent forms, subjects will be assigned to experimental group (rTMS at HT7), positive-control group (rTMS at r-DLPFC), and waiting-list control group (no treatment) randomly. Each subject will receive the different stimulation forms, even no stimulating, whereas all subjects will be aware of the grouping.

### 2.13 Statistical analysis

Data analysis will be based on intention-to-treat analysis and/or per-protocol analysis principle in the light of data missingness. We will also evaluate the group effect by comparing the analysis results between the above 2 data sets to evaluate robustness of our analytical results.

All data will be analyzed using SPSS23.0 (v23.0; SPSS Inc, Chicago, IL) by 3rd-party statistician. A *P* value <.05 will be considered as significant. Descriptive statistics will be performed with mean, standard deviation, maximum, minimum, and so on by groups and time.

Baseline characteristics of patients will be summarized by groups and compared with Chi-squared for categorical variables, and analysis of variance (ANOVA) (normal distributions) or Kruskal–Wallis test (non-normal distributions) for the continuous variables. Primary and secondary outcomes according to the measurement times are applicable to univariate repetitive measurement deviation analysis or ANOVA or Kruskal–Wallis test. If there is a violation of distribution assumption, appropriate transformation will be used.

#### 2.13.1 Primary outcomes

We will use univariate repetitive measurement deviation analysis with ISI scores and RMT before and the 10th day of treatment, 30th day of follow-up as the outcomes. A significant effect of group indicates that the ISI scores and RMT are different between groups after intervention.

#### 2.13.2 Secondary outcomes

Continuous outcome variables including Pittsburgh sleep quality index scores, Beck Depression Inventory-2nd edition scores, Beck anxiety inventory scores and Montreal Cognitive Assessment scores before and the 10th day of treatment will be analyzed with ANOVA or Kruskal–Wallis test. Categorical outcome variables in the patients’ diary, Chi-squared test will be used for comparison between groups.

#### 2.13.3 Safety evaluation

Adverse events recorded in the patients’ diary will be analyzed as a multiply variable. The number and percentage of patients with AE will be calculated and compared using Chi-squared test.

### 2.14 Quality control

The reasons of dropouts or withdrawals throughout the treatment and follow-up periods will be fully recorded. To ensure trial quality, the quality monitors will verify all the process details at regular intervals and check the authenticity of the data. Moreover, a 3rd party, scientific research department of Shaanxi Province Hospital of Traditional Chinese Medicine, will be invited to manage the data independently.

Since differences among physicians can cause bias, the evaluation of the rating scales in every time will be executed by one assigned physician who has been trained with the same criterion of evaluating. Two physicians responsible for treatment will receive special training about the study and manipulations including TMS use and locations of acupoints before recruitment. To record the attendance and compliance, we make record cards for patients which cover date of treatment, personal information, and their signatures after every treatment.

## 3 Discussion

The CI is a persistent insomnia disease, which the course of the disease is >3 months and frequency more than 3 times/wk.^[8]^ The onset of CI is affected by many factors involving age, gender, medical history, stress events, personality traits, inheritance, mental disorders, physical diseases, and so on.^[25]^ Refer to the diagnostic criteria of CI, CI patients who are long-term subjected to insomnia problem, will bring direct or indirect harm to the society. Therefore, according to patients symptoms combined with rating scale, we hope to obtain high quality study data from this preliminary clinical trial that could provide reliable basis to future multicenter research.

The rTMS at r-DLPFC which is chosen as a positive controlled therapy for CI in this study has been recommend by experts consensus.^[12]^ As a noninvasive stimulation technique, rTMS can test changes in motor evoked potential, brain electrical activity, cerebral blood flow, metabolism, and brain functional status from the changes in neural activity.^[27]^ Besides, DLPFC plays an important role in our cognition, decision, and memorization, which is always as an effective stimulating target for the treatment of insomnia and depression, especially low-frequency (1 Hz) rTMS at DLPFC.^[12]^ However, rTMS on the static head directly might be limited with some potential risks like scalp tingling and burning, hearing impairment, even seizure, especially for the ones with metal implants in skull or body.^[28]^ Moreover, it is inconvenient for insomnia patients to receive continuing care in daily life with rTMS at DLPFC. For this, rMS at acupoint on CI may be a promising alternative therapy. Acupoints have been widely used and received in clinical practice in China.^[29]^ Patients in China are usually familiar with acupuncture, and expect to receive acupuncture treatments. Thus, since acupuncture is often regarded as an invasive operation,^[30]^ it is difficult to replicate its manipulations in clinic and design a nonpenetrating sham acupuncture in trial.^[31]^ Hence, aim to improve subjects compliance and achieve acupoint effect, we choose rMS at HT7 acupoint in the experimental group in a high-quality randomized controlled trial. Although studies have showed the efficacy of rTMS for CI,^[32,33]^ it is still lack rMS at acupoint randomized controlled trial, as well as the relevant mechanism research.

Our protocol is just a pilot study with several limits including the small sample size, open-blinding, and short period of observation time. However, we try our best to attempt to minimize the biases that may influence study results. Finally we intend to demonstrate the hypothesis that rMS at HT7 acupoint as an alternative may be validated for CI.

## Author contributions

Jie Yuan and Jie Chen designed this study together. Jie Yuan and Hui Wang drafted the protocol. Zhaoxin Wan performed the statistical analysis. Yaling Lei was responsible for the writing revision. All authors read and approved the final manuscript.

**Data collection:** Pei Wang, Fan Luo, Yuan Wang.

**Formal analysis:** Zhaoxin Wan, Yuan Zhao.

**Funding acquisition:** Jie Chen, Jie Yuan.

**Investigation:** Yue Cao.

**Methodology:** Jie Yuan, Yuan Zhao.

**Project administration:** Zucheng Han, Dongling Liu.

**Writing – original draft:** Jie Yuan and Jie Chen.

**Writing – review & editing:** Yaling Lei, Jie Chen.
